# Activation of the hypothalamic–pituitary–adrenal axis by exogenous and endogenous GDF15

**DOI:** 10.1073/pnas.2106868118

**Published:** 2021-06-29

**Authors:** Irene Cimino, Hanna Kim, Y. C. Loraine Tung, Kent Pedersen, Debra Rimmington, John A. Tadross, Sara N. Kohnke, Ana Neves-Costa, André Barros, Stephanie Joaquim, Don Bennett, Audrey Melvin, Samuel M. Lockhart, Anthony J. Rostron, Jonathan Scott, Hui Liu, Keith Burling, Peter Barker, Menna R. Clatworthy, E-Chiang Lee, A. John Simpson, Giles S. H. Yeo, Luís F. Moita, Kendra K. Bence, Sebastian Beck Jørgensen, Anthony P. Coll, Danna M. Breen, Stephen O’Rahilly

**Affiliations:** ^a^Metabolic Research Laboratories, Wellcome Trust–Medical Research Council Institute of Metabolic Science, University of Cambridge, Cambridge CB2 0SL, United Kingdom;; ^b^Internal Medicine Research Unit, Pfizer Inc., Cambridge, MA 02139;; ^c^Global Obesity and Liver Disease Research, Novo Nordisk A/S, DK-2760 Maaloev, Denmark;; ^d^Department of Pathology, University of Cambridge, Cambridge CB2 1QP, United Kingdom;; ^e^Innate Immunity and Inflammation Laboratory, Instituto Gulbenkian de Ciência, 2780-156 Oeiras, Portugal;; ^f^Biostatistics, Early Clinical Development, Pfizer Inc., Cambridge, MA 02139;; ^g^Translational and Clinical Research Institute, Newcastle University, Newcastle upon Tyne NE2 4HH, United Kingdom;; ^h^Integrated Critical Care Unit, Sunderland Royal Hospital, South Tyneside and Sunderland NHS Foundation Trust, Sunderland SR4 7TP, United Kingdom;; ^i^Kymab Ltd., Cambridge CB22 3AT, United Kingdom;; ^j^Core Biochemical Assay Laboratories, Cambridge University Hospitals NHS Foundation Trust, Cambridge CB2 0QQ, United Kingdom;; ^k^Molecular Immunity Unit, Department of Medicine, University of Cambridge, Cambridge CB2 0QH, United Kingdom;; ^l^Cambridge Institute of Therapeutic Immunology and Infectious Diseases, University of Cambridge, Cambridge CB2 0AW, United Kingdom;; ^m^Cellular Genetics, Wellcome Sanger Institute, Hinxton CB10 1RQ, United Kingdom;; ^n^Instituto de Histologia e Biologia do Desenvolvimento, Faculdade de Medicina, Universidade de Lisboa, 1649-004 Lisboa, Portugal

**Keywords:** gdf15, corticosteroids, stress, toxins, adrenal

## Abstract

An acute increase in the circulating concentration of glucocorticoid hormones is essential for the survival of severe somatic stresses. Circulating concentrations of GDF15, a hormone that acts in the brain to reduce food intake, are frequently elevated in stressful states. We now report that GDF15 potently activates the hypothalamic–pituitary–adrenal (HPA) axis in mice and rats. A blocking antibody to the GDNF-family receptor α-like receptor completely prevented the corticosterone response to GDF15 administration. In wild-type mice exposed to a range of stressful stimuli, circulating levels of both corticosterone and GDF15 rose acutely. In the case of *Escherichia coli* or lipopolysaccharide injections, the vigorous proinflammatory cytokine response elicited was sufficient to produce a near-maximal HPA response, regardless of the presence or absence of GDF15. In contrast, the activation of the HPA axis seen in wild-type mice in response to the administration of genotoxic or endoplasmic reticulum toxins, which do not provoke a marked rise in cytokines, was absent in *Gdf15*^−/−^ mice. In conclusion, consistent with its proposed role as a sentinel hormone, endogenous GDF15 is required for the activation of the protective HPA response to toxins that do not induce a substantial cytokine response. In the context of efforts to develop GDF15 as an antiobesity therapeutic, these findings identify a biomarker of target engagement and a previously unrecognized pharmacodynamic effect, which will require monitoring in human studies.

## Significance

GDF15, a hormone produced by a wide variety of cells undergoing different types of stress, acts on a receptor in the brain where it transmits signals that are perceived by the organism as aversive. We now report an action of GDF15, whereby it activates the endocrine stress response and increases circulating levels of the principal glucocorticoid (a “stress” steroid). By studying mice genetically deficient in GDF15, we also demonstrate that GDF15 is a key signal through which damage due to toxic chemicals activates the steroid stress response. GDF15 is currently being explored as an antiobesity drug and examination of the degree and duration of the steroid effect will need to be incorporated into any human trials.

The activation of the hypothalamic–pituitary–adrenal (HPA) axis, which results in an increase in circulating glucocorticoids, is a stereotypical response to a wide range of stressful stimuli. Through their antiinflammatory, metabolic, and vasomotor effects, glucocorticoid hormones assist the organism in withstanding life-threatening challenges (1).

While the HPA axis responds acutely to a range of external threats perceived by dedicated sensors, it is also responsive to the status of the internal milieu. Thus, in the context of infection or severe tissue damage, proinflammatory cytokines, such as TNFα/β, IL-1, and IL-6, activate the axis (reviewed in ref. 2). During starvation, a fall in circulation leptin concentrations is sensed by the hypothalamus and conveyed to the corticotropin-releasing hormone (CRH) neurons, which initiate the HPA response (3). More recently it has been recognized that fibroblast growth factor 21 (FGF21), a largely hepatically derived hormone, the levels of which are increased by prolonged fasting, can also activate the HPA axis (4).

Threats to the organism can also arise from noninfectious agents, such as ionizing radiation, temperature, hypoxia, or the accidental ingestion of, or envenomation by, toxic chemicals. Exposure to the latter is likely to have played an important evolutionary role as a surprisingly large percentage of the genome of metazoan organisms, the so-called “chemical defensome,” is devoted to genes concerned with the recognition, inactivation, and disposition of xenobiotic substances (5). Earlier literature contains several reports of increases in circulating glucocorticoid levels in rodents occurring in response to a range of toxins, including honey bee and snake venom (6, 7), cyanide (8), and purified diphtheria toxin (8). More recently, genotoxins such as cisplatin have been shown to activate the HPA axis in dogs and rats (9, 10).

Unbiased transcriptomic screens of cellular responses to chemical toxins have frequently identified the transforming growth factor beta (TGF-β) superfamily member, growth differentiation factor 15 (*Gdf15*), as one of the most highly up-regulated genes (11, 12). GDF15 is ubiquitously produced in the body, with circulating concentrations rising rapidly upon exposure to a wide variety of stressors (12, 13).

GDF15 signals via a heterodimeric receptor, GDNF-family receptor α-like (GFRAL)-RET, localized specifically in the brainstem (14–17). To date, reports of the central actions of GDF15 in mammals have largely focused on regulation of food intake anorexia, weight loss (18–20), emesis (21), pica (22), delayed gastric emptying (23, 24), and conditioned aversion (25). Recent data indicate that GDF15 administration also reduces physical activity in mice (26). This range of actions would be consistent with GDF15 playing a role in signaling the presence of chemical threats to the organism which might be mitigated by reduced rate of exposure to, or expulsion of, ingested toxins and the promotion of their avoidance in future.

We undertook a set of experiments to examine whether GDF15 might be involved in the HPA response to stress; possibly synergizing with cytokines in the case of infections and/or playing a more prominent role in the response to chemical stressors (27). As examples of the latter, we chose the genotoxin, cisplatin, known to elevate circulating levels of both corticosterone (9, 10) and GDF15 (21, 22) and the well-established inducer of endoplas mic reticulum (ER) stress, tunicamycin. ER stress is mechanistically distinct from genotoxicity and its effects to increase GDF15 expression and secretion are well established (20, 28).

We now report that GDF15, acting through its receptor, GFRAL, is a powerful activator of the HPA axis. While its endogenous concentration rises in response to infectious stimuli, GDF15 is not necessary for the activation of the HPA axis. In contrast, the robust HPA activation that results from exposure to chemical stressors, which do not cause a substantial rise in proinflammatory cytokines, is highly dependent on GDF15.

## Results

### Exogenous GDF15 Activates the HPA Axis in a GFRAL-Dependent Manner.

**Fig. 1.**
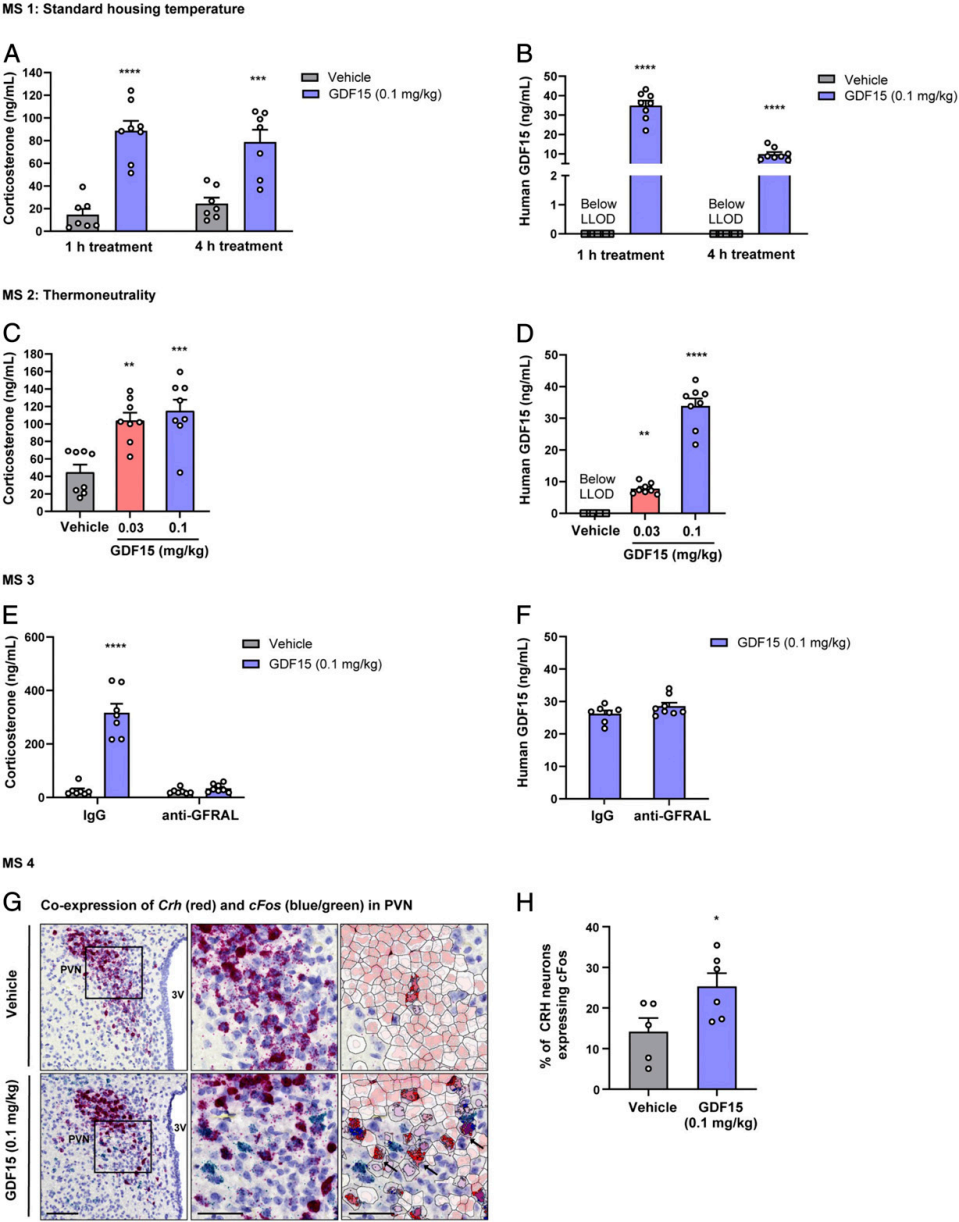
Acute administration of human recombinant GDF15 activates the HPA axis in mice. (*A* and *B*) Mouse study 1 (MS1) at standard housing condition: acute effect of human recombinant GDF15 administration on (*A*) endogenous corticosterone and (*B*) human GDF15 plasma concentration at 1 h and 4 h post-GDF15 treatment. (*C* and *D*) MS2 at thermoneutral housing condition: acute effect of two doses of human recombinant GDF15 on (*C*) plasma corticosterone and (*D*) human GDF15 concentrations after 1-h treatment (0.03 and 0.1 mg/kg). (*E* and *F*) MS3: (*E*) Corticosterone serum level in anti-GFRAL- and IgG control-treated mice with or without human recombinant GDF15 administration. (*F*) Human GDF15 serum concentration in GFRAL blocking antibody (anti-GRFAL) and IgG groups treated with human recombinant GDF15. (*G* and *H*) MS4: (*G*) In situ hybridization analysis of *Crh* mRNA (red), *c-Fos* mRNA (blue-green), and hematoxylin counterstain for nuclei at the level of the PVN. *Left*, representative images of coronal sections of vehicle (*Upper Left*) and GDF15-treated mice (*Lower Left*). (Scale bar, 50 μm.) 3V, third ventricle. *Middle*, higher magnification of the PVN showing coexpression of *Crh* and *c-Fos* dots in vehicle (*Upper Middle*) and GDF15 treated (*Lower Middle*). (Scale bar, 100 μm.) *Right*, automated quantification of *Crh-* and *c-Fos*-positive cells in vehicle (*Upper Right*) and GDF15-treated mice (*Lower Right*) (Scale bar, 100 μm.) Black arrows indicate double-labeled cells. Cell nucleus color in the overlay (*Right*) represents the cell classification (muted red for *Crh*-positive cells, bright red for dual-positive cells), while nucleus color intensity correlates with spot counts per cell. Bright blue spots represent *c-Fos* spot assigned to a *Crh*-positive cell. (*H*) Percentage of *Crh*-positive cells that were also *c-Fos* positive. Data are expressed as mean ± SEM, *n* = 6 to 8 per group. *P < 0.05, **P < 0.01, ***P < 0.001, and ****P < 0.0001, for MS1, -2, and -3, data were analyzed by ANOVA and for MS4, by unpaired Student’s *t* test.

We sought to determine whether GDF15 activated the HPA axis in mice. We used recombinant human GDF15 at a dose (0.1 mg/kg) that has previously been shown to significantly reduce food intake (20). Mice housed at standard temperature were injected with GDF15, or vehicle, with subsequent blood samples taken at 1 and 4 h postinjection. Corticosterone concentrations increased ∼3.5 fold at 1 h and were still significantly elevated at 4 h post-GDF15 treatment (Fig. 1*A*). The peak circulating concentrations of human GDF15 achieved in the mice (Fig. 1*B*) were comparable to those reported in states such as severe sepsis (29). This effect was confirmed in both sexes (*SI Appendix*, Fig. S1 *A* and *B*) and discernible as early as 30 min postinjection (*SI Appendix*, Fig. S1*C*). Similar results were obtained when studies were performed at thermoneutrality to avoid any possible effect of cold stress from standard housing temperature (Fig. 1 *C* and *D*). We also studied a lower dose of GDF15 (0.03 mg/kg) under the same conditions. This produced circulating concentrations of human GDF15 (Fig. 1*D***)** similar to that reported in patients with cancer and heart failure (30, 31). This dose also resulted in a significant increase in circulating corticosterone, which was almost as marked as that seen with the higher dose (Fig. 1*C*). The corticosterone response to GDF15 was not attributable to any acute reduction in circulating leptin concentration, which was not significantly changed at 1 h (*SI Appendix*, Fig. S1*D*).

To determine whether the corticosterone response to GDF15 was specifically mediated through its hindbrain receptor, we undertook experiments using a neutralizing anti-GFRAL antibody, having validated its efficacy on classical GDF15 responses in mice. At a dose of 20 mg/kg, the antibody blocked GDF15-induced food intake reduction and body weight loss (*SI Appendix*, Fig. S1 *E* and *F* and *Methods*). Furthermore, the anti-GFRAL antibody completely prevented GDF15-induced corticosterone concentrations while a control isotype antibody had no effect (Fig. 1*E*). The concentrations of human circulating GDF15 achieved in the study did not differ between the anti-GFRAL group and control isotype (Fig. 1*F*).

As the increase of circulating glucocorticoids is centrally driven by a discrete population of CRH neurons in the paraventricular nucleus (PVN) of the hypothalamus and GDF15 administration has been shown to activate this region (32), we used dual in situ hybridization (RNAScope) to examine the expression of *c-Fos* and *Crh* in the mouse hypothalamus under basal conditions and in response to the peripheral administration of GDF15. The mice were killed at 1 h postinjection and hypothalamic sections were prepared and stained. GDF15 significantly increased the proportion of *Crh* neurons expressing *c-Fos*, thus confirming activation of *Crh*-expressing neurons (Fig. 1 *G* and *H*).

**Fig. 2.**
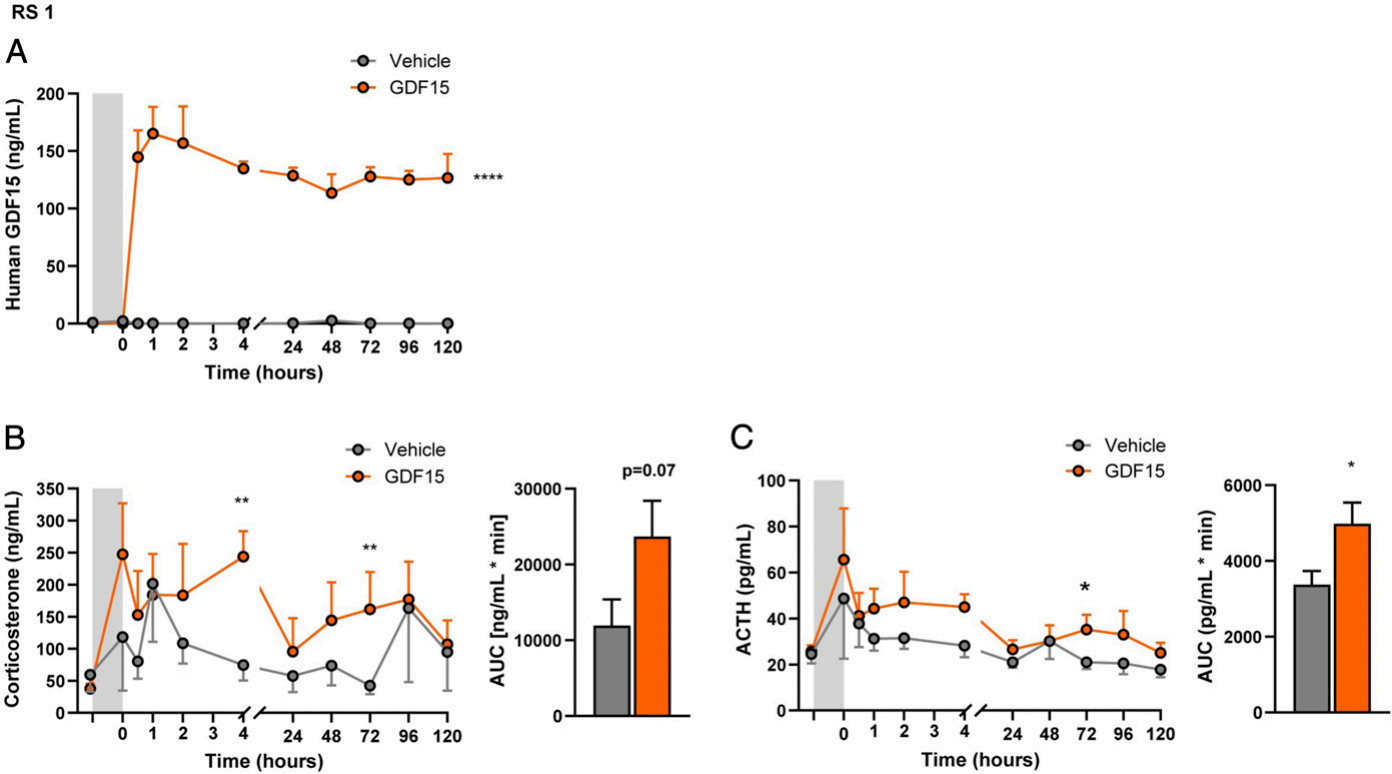
Chronic infusion of human GDF15 activates the HPA axis in rats. Rat study 1 (RS1): (*A*) Plasma concentration time course of human GDF15 in rats with continuous intravenous infusion of vehicle or human GDF15. Gray shade indicates the period with bolus infusion of 0.24 mg/kg/h followed by a period with maintenance infusion of 0.04 mg/kg/h. (*B*) Plasma concentration time course of endogenous corticosterone in rats in response to continuous intravenous infusion of vehicle buffer or human GDF15. Plasma corticosterone levels are expressed as the area under the curve (AUC) calculated from time point zero to the termination of the study. (*C*) Plasma concentration time course of endogenous ACTH in rats in response to continuous intravenous infusion of vehicle or human GDF15. Plasma corticosterone levels expressed as the AUC calculated from time point zero to the termination of the study. Data are expressed as mean ± SEM, *n* = 5 to 6. *P < 0.05 **P < 0.01, ****P < 0.0001 by repeated measurement model with baseline assessment as a covariate.

The effect of chronic exposure to GDF15 was also investigated in cannulated rats, which allowed serial sampling and measurement of adrenocorticotropic hormone (ACTH) as well as corticosterone, with minimal handling stress. GDF15 (or vehicle) was administered as an acute bolus over 1 h followed by 5 d of sustained infusion (*SI Appendix*, Fig. S2*A*). After 1 h of GDF15 infusion, the concentrations of human GDF15 peaked and were sustained at 100 to 150 ng/mL throughout the study (Fig. 2*A*). The efficacy of human recombinant GDF15 was validated by the suppression of food intake by up to 70% accompanied by a 16% reduction of body weight (*SI Appendix*, Fig. S2 *B* and *C*). Compared to vehicle-treated animals, corticosterone levels rose significantly at 4 h. With chronic infusion, corticosterone levels declined from their initial peak but tended to remain higher in the GDF15-infused animals throughout all of the infusion period (Fig. 2*B*). Supporting a central rather than peripheral effect of GDF15, we found that ACTH levels were significantly higher than the vehicle-infused animals throughout the experiment (Fig. 2*C*). This is noteworthy as corticosterone exerts strong negative feedback on CRH release. The maintenance of elevated ACTH levels under circumstances where corticosterone levels are the same or slightly higher than vehicle-infused animals indicates a sustained action of GDF15 to stimulate the axis.

We then proceeded to perform a series of experiments designed to examine the role of endogenous GDF15 as a stimulus to the HPA axis in response to a variety of stressors.

### Infection-Induced Activation of the HPA Axis Does Not Require GDF15.

It is well established that proinflammatory cytokines can activate the HPA axis in the context of systemic infections. Circulating GDF15 levels are also elevated in response to infectious stimuli in humans and mice (33, 34). We therefore wished to test whether a rise in circulating GDF15 in this context might contribute to the activation of the HPA axis effects. We did this by examining the HPA response in wild-type and *Gdf15*-deficient mice (*Gdf15*^*−/−*^) to two different infection-related stimuli: 1) lipopolysaccharide (LPS)-induced endotoxemia and 2) systemic infection with *Escherichia coli*.

**Fig. 3.**
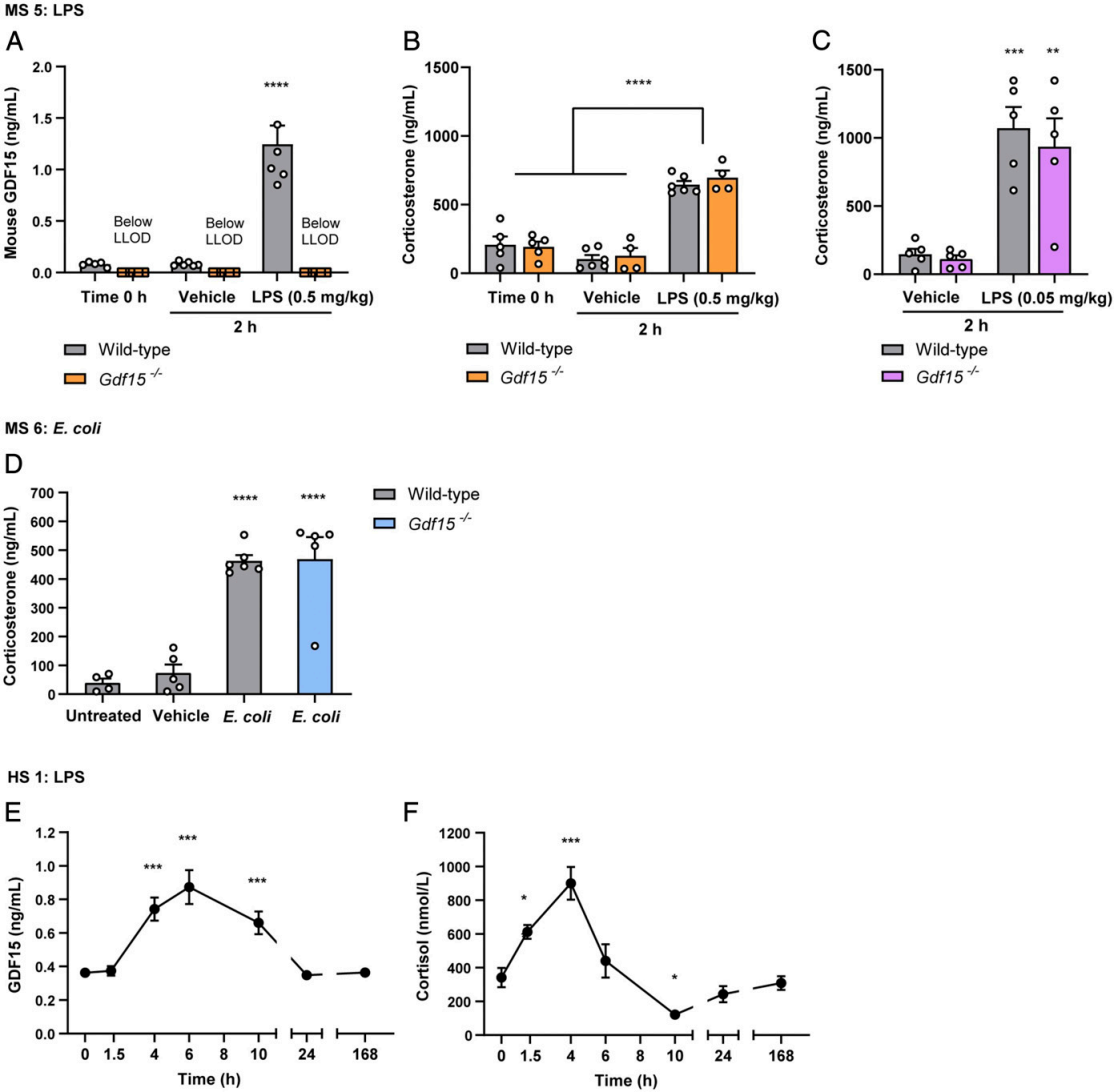
GDF15 is not necessary in mediating the LPS-induced rise in glucocorticoids in mice and humans. MS5: (*A*) Mouse GDF15 and (*B*) corticosterone serum concentrations at baseline (time = 0) and 2 h after LPS (0.5 mg/kg) or vehicle control injection in wild-type and *Gdf15*^*−/−*^ mice. (*C*) Corticosterone serum concentration 2 h after LPS (0.05 mg/kg) or vehicle control injection in wild-type and *Gdf15*^*−/−*^ mice. (*D*) MS6: Corticosterone serum concentration 4 h after *E. coli* infection in wild-type and *Gdf15*^*−/−*^ mice. (*E* and *F*) Human study 1 (HS1): Time course of (*E*) GDF15 and (*F*) cortisol serum levels at baseline (time = 0) and after 2 ng/kg bolus intravenous infusion of LPS in healthy human subjects. For MS5 and MS6 data are expressed as mean ± SEM, *n* = 4 to 6 per group. *P < 0.05, **P < 0.01, ***P < 0.001, ****P < 0.0001 by ANOVA. For HS1 data are expressed as mean ± SEM, *n* = 11. *P < 0.05, ***P < 0.001 by one-way repeated measures with post hoc Dunnett’s test to compare each time point with baseline.

The administration of LPS at 0.5 mg/kg produced a robust increase in circulating GDF15 (Fig. 3*A*). LPS also induced a rise in corticosterone (Fig. 3*B*), which was similar in wild-type or *Gdf15*^*−/−*^ mice at the time point analyzed (2 h). As expected, GDF15 (*SI Appendix*, Fig. S3*A*), corticosterone (*SI Appendix*, Fig. S3*B*), and cytokine levels were markedly increased by LPS injection at all time points analyzed (*SI Appendix*, Table S1) to an extent that was independent of GDF15 status. In the experiments undertaken with a lower dose of LPS (0.05 mg/kg) corticosterone responses were again very similar in wild-type and *Gdf15*^*−/−*^ mice (Fig. 3*C*).

In independent experiments *Gdf15*^*−/−*^ mice or their wild-type controls were injected with either *E. coli* or vehicle as previously described (35). The rise in corticosterone occurred at 4 h but was not different between the two genotypes (Fig. 3*D*).

Consistent with a largely GDF15-independent effect of infection on the HPA axis, in human participants injected with low-dose endotoxin the acute rise in cortisol as well as inflammatory factors preceded the rise in GDF15 (Fig. 3 *E* and *F* and *SI Appendix*, Fig. S3 *C*–*E*).

Thus, although GDF15 levels increased markedly in response to infection-related stimuli, this was not necessary for the activation of the HPA axis. It appears likely that proinflammatory cytokines such as IL-6, IL-1β, and TNF-α, all of which were significantly increased post-LPS challenge, play the dominant role under these circumstances. Indeed, the levels of corticosterone reached with these powerful infection-related stimuli may be close to maximal, thereby obscuring any effects of GDF15.

### The Glucocorticoid Response to Chemical Toxins Is GDF15 Dependent.

Other than infections, there are a range of potentially life-threatening stressors to which an organism may be exposed (36) and many of these have been reported to induce the expression of GDF15 (11). We chose to examine the role of GDF15 in the activation of the HPA in response to exposure to chemical toxins. We selected cisplatin as a genotoxin and tunicamycin as an inducer of ER stress.

**Fig. 4.**
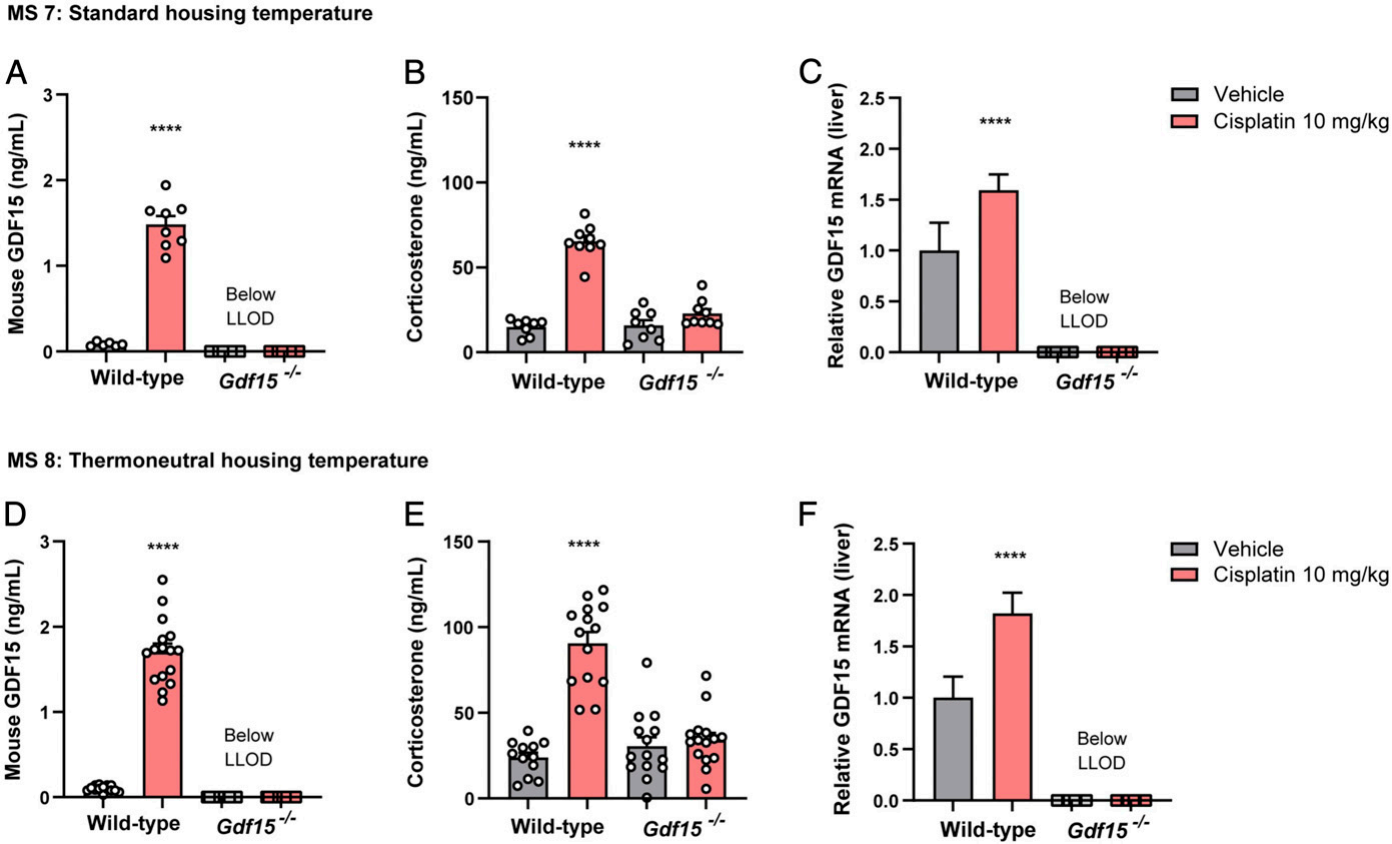
GDF15 is necessary to mediate corticosterone rising upon cisplatin administration. MS7 at standard housing temperature: (*A*) Mouse GDF15 and (*B*) corticosterone plasma concentration and (*C*) *Gdf15* mRNA expression in the liver after 6-h cisplatin administration (10 mg/kg) in wild-type and *Gdf15*^*−/−*^ mice. (*D–F*) MS8 at thermoneutral housing condition: (*D*) Mouse GDF15 and (*E*) corticosterone plasma concentration and (*F*) *Gdf15* mRNA expression in the liver after 6-h cisplatin injection (10 mg/kg) in wild-type and *Gdf15*^*−/−*^ mice. Data are expressed as mean ± SEM, For MS7, *n* = 8 to 9 per group, MS8, *n* = 13 to 16 per group, ****P < 0.0001 by ANOVA.

Previous studies have reported that cisplatin significantly increases circulating GDF15 levels in humans and in preclinical models (17, 21, 22) We first studied the effects of cisplatin on the HPA axis in *Gdf15*^*−/−*^ mice and wild-type littermates. Independent studies were undertaken at standard and thermoneutral housing temperatures. Under both conditions, as expected, cisplatin caused a robust rise in circulating GDF15 and *Gdf15* mRNA in the liver in wild-type but not in *Gdf15*^*−/−*^ mice (Fig. 4 *A*, *C*, *D,* and *F*). Notably, corticosterone levels increased approximately threefold at 6 h in the wild-type mice and not in the *Gdf15*^*−/−*^ mice (Fig. 4 *B* and *E*). In contrast to infectious models, cisplatin does not increase circulating levels of proinflammatory markers during the time course of these experiments (21).

GDF15 is known to be potently induced by ER stress (20, 28, 37). We chose to use tunicamycin, a bacterially derived inhibitor of *N*-linked protein glycosylation, as an ER stressor for in vivo studies in mice at standard and thermoneutral conditions.

**Fig. 5.**
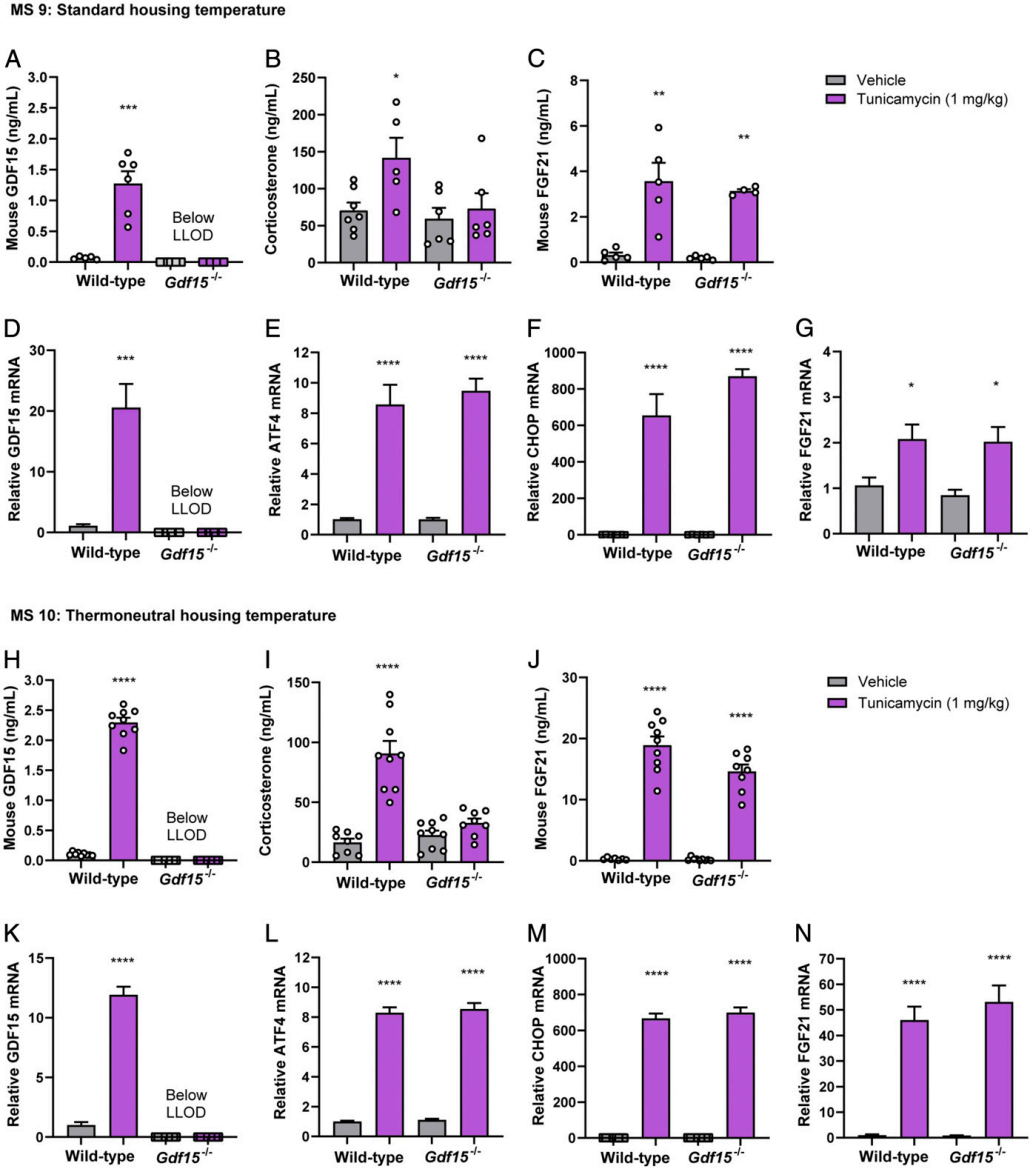
GDF15 is partially needed to mediate corticosterone rising upon tunicamycin-induced ER stress. (*A–G*) MS9 at standard housing temperature: (*A*) Mouse GDF15, (*B*) corticosterone, and (*C*) FGF21 serum levels after 6-h tunicamycin injection in wild-type and *Gdf15*^*−/−*^ mice. mRNA expression of (*D*) *Gdf15*, (*E*) *Atf4*, *(F*) *Chop*, and (*G*) *Fgf21* in the liver after 6-h tunicamycin injection in wild-type and *Gdf15*^*−/−*^ mice. (*H–N*) MS10 at thermoneutral housing condition: (*H*) Mouse GDF15, (*I*) corticosterone, and (*J*) FGF21 plasma levels after 6-h tunicamycin injection in wild-type and *Gdf15*^*−/−*^ mice. (*K*) *Gdf15*, (*L*) *Atf4*, (*M*) *Chop*, and (*N*) *Fgf21* mRNA expression in the liver after 6-h tunicamycin injection. Data are expressed as mean ± SEM, *n* = 5 to 9 per group. *P < 0.05, **P < 0.01, ***P < 0.001, ****P < 0.0001 by ANOVA.

Six hours after tunicamycin treatment, we observed an increase in circulating GDF15 (Fig. 5 *A* and *H*) and in the hepatic expression of *Gdf15* mRNA in the wild-type mice (Fig. 5 *D* and *K*). As expected, we also observed an increase in the hepatic levels of mRNA encoding the classical ER stress markers *Atf4* and *Chop* in both *Gdf15*^*−/−*^ and wild-type mice (Fig. 5 *E*, *F*, *L,* and *M*). In contrast to cisplatin, tunicamycin treatment did result in a rise in the circulating levels of some proinflammatory cytokines, but this was limited to a minority of those assayed and modest in extent, being far less than the rise observed in response to LPS. At the 6-h time point, when the HPA axis was assessed, only IL-12 was elevated in both wild-type and *Gdf15*^−/−^ mice (*SI Appendix*, Table S2), with no substantial induction of TNF-a, IL-6, or IL-1β, proinflammatory cytokines previously shown to activate the HPA axis (38, 39).

We proceeded to study the effects of tunicamycin on the HPA axis in *Gdf15*^*−/−*^ and wild-type mice. Six hours after tunicamycin, corticosterone levels were elevated threefold in the wild-type mice, and the response was markedly attenuated in the *Gdf15*^*−/−*^animals (Fig. 5 *B* and *I*).

Hepatic ER stress is also known to increase the expression and secretion of FGF21, an endocrine member of the fibroblast growth factor (FGF) family, which is known to act in the brain where, among other effects, it can also activate the HPA axis. Since tunicamycin-induced corticosterone is partially GDF15 dependent, we measured circulating FGF21 level in mice after tunicamycin injection. Tunicamycin increased circulating FGF21 (Fig. 5 *C* and *J*) and also *Fgf21* mRNA levels in the liver (Fig. 5 *G* and *N*).

**Fig. 6.**
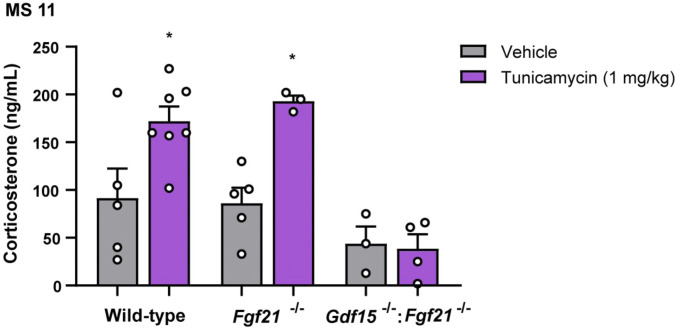
GDF15 and FGF21 show a synergic action in mediating tunicamycin-induced corticosterone rising. MS11: Corticosterone serum levels in wild-type, *Fgf21*^*−/−*^, and *Gdf15*^*−/−*^*:Fgf21*^*−/−*^ mice 6 h after tunicamycin administration compared to vehicle control. Data are expressed as mean ± SEM, *n* = 3 to 7 per group. *P < 0.05 by ANOVA.

To examine the relative importance of GDF15 and FGF21 in mediating the effect of tunicamycin-induced ER stress on the HPA axis, we studied mice lacking FGF21 (*Fgf21*^−/−^) as well as mice doubly null for *Fgf21* and *Gdf15* (*Gdf15*^*−/−*^*:Fgf21*^*−/−*^). *Fgf21*^−/−^ mice responded to tunicamycin with a corticosterone response that was indistinguishable from the wild type (Fig. 6). In contrast, the double-deficient mice showed no discernible corticosterone response to tunicamycin (Fig. 6). We conclude that while FGF21 may make a small contribution to the effects of ER stress on the HPA axis, GDF15 is clearly the dominant signal.

## Discussion

Since its identification in 1997 as a novel endocrine member of the TGF-β superfamily expressed in activated macrophages (40), circulating levels of GDF15 have been reported to be elevated in a broad spectrum of conditions, including many disease states (reviewed in refs. 12 and 13). GDF15 expression can be induced in many, perhaps all, tissues in response to a variety of different stressful stimuli (reviewed in refs. 12 and 13). The identification of a brainstem-restricted receptor and of a wide range of centrally controlled behavioral and gastrointestinal responses have led to the suggestion that GDF15 may be a “sentinel” hormone. The role of such a signal would include limiting systemic exposure to recently ingested toxins (through its effect on vomiting and pica) and, through its induction of conditioned aversion, promoting the avoidance of future exposures to agents, which have previously led to cellular stress in the host (12).

We now describe another important action of GDF15, namely the activation of the HPA axis. This occurs acutely at levels of circulating GDF15, similar to those found naturally in response to various stressors and illnesses, and requires GFRAL (41, 42). Hindbrain neurons expressing GFRAL project to the parabrachial nucleus (32, 43) and from that nucleus, there are well-established projections to the PVN (44) where CRH-expressing neurons were activated by GDF15. Endogenous GDF15 was not required for the HPA response to infection-related stimuli but was essential for the response to the administration of toxins of two different classes. It is worthy of note that, consistent with the acutely life-threatening nature of systemic infections, peak corticosterone levels occurring as a result of infection-related stimuli are substantially higher than those found after the administration of the toxins or after the administration of high doses of GDF15. It seems reasonable to speculate that the cytokine “storm,” which occurs in response to LPS or *E. coli* injection, results in a maximal or near-maximal activation of the HPA axis, rendering any additional input to the axis from signals emanating from GDF15-activated neurons in the hindbrain redundant under those circumstances.

GDF15 levels rise in response to a very broad range of injurious agents, including ionizing radiation (45), hypoxia (46), intense physical activity (47), and a wide range of chemical toxins all of which can activate a stress response and many of which also elevate GDF15 (11, 12) but not all of which cause an acute cytokine response (21). We examined two agents known to increase circulating GDF15, one of which is genotoxic (cisplatin) (21, 22) and the other, tunicamycin, which produces marked ER stress by perturbing protein folding (20, 28). Both agents resulted in a marked increase in both GDF15 and corticosterone. Cisplatin has previously been reported to activate the HPA axis in rats and dogs (9, 10). There is one report in humans (48), which describes the opposite effect but blood sampling in this study was limited to the first 6 h after drug administration and to a small number of patients. To our knowledge, there are no previous reports of the effects of systemically administered ER stressors on the HPA axis.

In a previous study we found no significant rise of proinflammatory cytokines in response to cisplatin (21). In our study the rise in corticosterone in response to cisplatin was absent in the mice lacking GDF15, whether housed at standard or thermoneutral temperatures. In the case of the ER stress response to tunicamycin there were small increases in some proinflammatory cytokines, but these were orders of magnitude lower than concentrations observed in response to infection-related stimuli, and in particular there was no substantial induction of TNF-a, IL-1β, and IL-6, the cytokines best described to induce the HPA axis.

Although FGF21 is also induced by ER stress (49), our experiments clearly demonstrated the dominance of GDF15 in mediating the HPA response to this type of cellular stress.

We have previously argued that GDF15 actions on the brain do not necessarily represent part of a homeostatic system controlling appetite and body weight but are more likely to represent an “allostatic” system involved in the response of the organism to major threats (20, 50). Activation of the HPA axis is a core feature of the response to a wide range of threats. The existence, in metazoan genomes, of a wide variety of highly conserved genes—the principal purpose of which appears to be to detect, inactivate, and dispose of xenobiotic substances—suggest that exposure to chemical stressors has been an omnipresent evolutionary pressure (51). It is therefore unsurprising to find that a system appears to have evolved to activate a key endocrine arm of the stress response after exposure to chemically activated cellular stress.

In addition to identifying a physiological/allostatic role for GDF15, the finding that it potently activates the HPA axis opens up a range of relevant questions in translational biomedicine. Could the effect in the HPA axis contribute to the development of diseases where levels of GDF15 are pathologically elevated? There are several human diseases where GDF15 concentrations are chronically elevated (52, 53). Many of these are associated with cachexia (54, 55), characterized by loss of fat and muscle mass (56). The effects of GDF15 on appetite and body weight have been well established, but glucocorticoids are powerfully antianabolic in skeletal muscle, raising the question of whether the loss of lean mass, particularly in skeletal muscle, could be attributed to the chronic activating effects of GDF15 on the HPA axis. It is worthy of note that we have recently reported an effect of glucocorticoids to suppress GDF15 levels, indicating that the interaction is bidirectional (57).

Our findings may also have implications for the development of GDF15 agonists as therapeutics for obesity and related metabolic disorders. Because of its action as an appetite suppressant, GDF15 is being explored as a potential therapy for obesity. Safety issues are central to the development of antiobesity therapeutics. As GDF15 analogs and agonists of its receptor are developed further as antiobesity agents, it may be relevant to determine the extent and durability of its actions on the HPA axis.

GDF15 has been reported to have antiinflammatory properties under various circumstances (35, 58–60). It has therefore been suggested that, in addition to reducing food intake and body weight, GDF15 might have additional potential therapeutic utility in ameliorating the inflammatory component of many cardiometabolic diseases (58, 61). The means by which GDF15 could exert its putative antiinflammatory actions have been obscure. Given the powerful antiinflammatory properties of corticosteroids, the additional action of GDF15, which we describe herein, is an obvious route through which such effects could be mediated. That said, other research has suggested that GDF15 can, under certain circumstances, be proinflammatory (62, 63) and Luan et al. recently reported that GDF15 promoted survival in the face of serious infection through effects on host tolerance rather than host defense without affecting the magnitude of the inflammatory response (34). This is an area that clearly requires further research.

Conversely, GDF15 antagonists are being developed to treat cachectic states, particularly those associated with cancer (64). In addition to improving appetite and food intake, GDF15 blockade might improve the anabolic state and increase muscle mass through reversing pathological hyperactivation of the HPA axis. GDF15 blockade is also being explored for its utility in reducing the side effects of cytotoxic chemotherapy particularly with platinum-based reagents (21). Further studies of the impact of GDF15 neutralization on the HPA in these circumstances are warranted.

In conclusion, the chemical defensome appears to have an endocrine circuit, in which GDF15 constitutes the afferent limb, transmitting signals of toxin-induced cellular stress to the brain, with glucocorticoids being the efferent hormonal output. This aspect of GDF15 action has important consequences for the evaluation of agonists and antagonists of the GDF15 signaling pathway as potential therapeutics for a range of human diseases.

## Methods

### Mice.

Briefly, adult wild-type and *Gdf15*^−/−^ mice were housed individually under a standard 12 h light:dark cycle (6:00 h:18:00 h) at standard housing temperatures (22 ± 1 °C) or thermoneutral conditions (27 ± 1 °C) with a humidity-controlled environment. Mice were given ad libitum access to food and water. Studies were carried out at three sites: Pfizer Inc, Cambridge, MA, the University of Cambridge, Cambridge, UK, and the Instituto Gulbenkian de Ciência (IGC), Portugal. Further details can be found in *SI Appendix*. At the University of Cambridge, the research was regulated under the Animals (Scientific Procedures) Act 1986 Amendment Regulations 2012 following ethical review by the University of Cambridge Animal Welfare and Ethical Review Body. At IGC, all animal studies were performed in accordance with Portuguese regulations and approved by the Instituto Gulbenkian de Ciência Ethics Committee and Direção Geral de Alimentação e Veterinária (reference A002.2015).

### Rat Study.

All animal experiments were carried out in accordance with the Danish Act on Experiments on Animals, and EU Directive 2010/63. A project license was issued by the national authority. All animal experiments were performed in accordance with relevant regulations and guidelines and approved by the Novo Nordisk Animal Welfare Body. Sprague–Dawley (SD) rats were purchased from Charles River Laboratories and were housed under standard conditions including a 12 h:12 h light:dark cycle, ∼21 °C, and water and food ad libitum. In rat study 1, single-housed SD rats (Charles River Laboratories) weighing ∼350 g were allowed to acclimatize in the BioDAQ (Research Diet, Inc.) food hopper/Accusampler (Verutech AB) catheter system for 8 to 9 d prior to surgery. Surgery was performed on day 1 and catheters (Tygon Microbore Tubing) were placed in the left carotid artery and the right jugular vein in isoflurane-anesthetized animals and filled with 100 IU/mL heparin in 0.9% NaCl to prevent clotting. After 5 to 6 d of additional acclimatization the study was initiated by a bolus infusion of vehicle or human recombinant GDF15 for 1 h (83 mL/kg/min ± 0.24 mg/kg/h GDF15) followed by a maintenance intravenous (iv) infusion throughout the rest of the study (73 mL/kg/min ± 0.04 mg/kg/h of GDF15). Arterial blood samples were collected automatically using the Accusampler system at prespecified time points (−65 min, 0 min, 30 min, 1 h, 2 h, 4 h, 24 h, 48 h, 72 h, 96 h, and 120 h) where time point zero is at the end of bolus infusion. A total of 200 µL of blood was collected and transferred to ice-cooled ethylenediaminetetraacetic acid (EDTA) tubes and immediately centrifuged for 2 min at 4,500 × *g* at 4 °C and kept at −80 °C until analysis. Plasma levels of GDF15 were measured using an in-house immunoassay. Plasma mouse corticosterone was measured using the mouse/rat corticosterone enzyme-linked immunosorbent assay (EIA) (Cat# AC14F1, IDS) and rat ACTH was measured by Milliplex kit (Cat# RPTMAG-86K, Merck-Millipore).

### Human Endotoxemia.

Human study 1 was performed in the Integrated Critical Care Unit at South Tyneside and Sunderland Foundation Trust, supervised by a critical care physician.

Ethical approval was granted by the Yorkshire and the Humber–South Yorkshire Research Ethics Committee (17/YH/0021), and the study was sponsored by Newcastle upon Tyne Hospitals National Health System (NHS) Foundation Trust. Ten healthy male volunteers (mean age 25 y, range 18 to 25) gave informed, written consent to receive intravenous administration of LPS (Cat# 94332B1, donated by the NIH) and injected intravenously as a bolus dose of 2 ng/kg. Baseline measurements were undertaken between 8:00 and 10:30. Human cortisol was measured using a chemiluminescent immunoassay (Cat# 313261, DiaSorin S.p.A.). Human GDF15 was measured using an electrochemiluminescent immunoassay on the MesoScale Discovery platform with antibodies and standards from R&D Systems Europe. Cytokine concentrations were measured using a human inflammatory cytokine cytometric bead array (CBA) (Cat# 551811, BD Biosciences).

### GDF15 Injection at Standard and Thermoneutral Housing Temperatures.

For mouse studies 1 and 2 (MS1 and MS2), human recombinant GDF15 (Cat# 4570, BioVision Inc.) was prepared in saline and administered via subcutaneous (s.c.) injection as a single dose in the morning (9 AM). For mouse studies, 12 to 14 human recombinant GDF15 (Cat# Qk017, Qkine) was administrated via s.c. injection as a single dose in the morning (9 AM). At 1 h and/or 4 h posthuman recombinant GDF15 treatment, mice were killed by CO_2_ inhalation and blood was collected via cardiac puncture. Blood was collected in EDTA tubes (Becton Dickinson and Company) and centrifuged to collect plasma, which was stored at −80 °C until analysis. For MS1 and MS2, plasma human GDF15 was measured using the human GDF15 Quantikine ELISA (R&D Systems) and plasma mouse corticosterone was measured using the mouse corticosterone ELISA (Cat# 55-CORMS-E01, ALPCO) as per manufacturer instructions. For MS12 to MS14, GDF15 was measured using the human GDF15 ELISA (Cat#DY957, R&D Systems, BioTechne) and plasma mouse corticosterone was measured using the mouse corticosterone EIA (Cat# AC14F1, IDS). Plasma leptin was measured using a Meso Scale Discovery two-plex mouse metabolic immunoassay kit (Cat# K15124C, Meso Scale Diagnostics).

### Anti-GFRAL Studies.

For mouse studies 3 and 15, the mice were injected intraperitoneally (i.p.) with anti-GFRAL monoclonal antibody (anti-GFRAL) (KyMab/Sanofi) at a dose of 20 mg/kg and with vehicle control. These injections were repeated twice at an interval of 1 d. The day after the last i.p. injection, either human recombinant GDF15 (0.1 mg/kg, Qkine) or vehicle control were injected s.c. Food intake and body weight were measured overnight and for 24 h. One week later, the same anti-GFRAL-IgG experimental paradigm was repeated, but this time, the day after the second injection the mice were injected with either GDF15 or vehicle control at 9 AM and killed 1 h after. Blood was collected and analyzed as described above.

### Brain Processing and RNAScope Analysis.

For mouse study 4, the mice were injected at 10:00 AM either with GDF15 (0.1 mg/kg Qkine) or vehicle control. One hour postinjection mice were killed, and brain tissue was collected and fixed. Simultaneous detection of mouse *Fos* and *Crh* was performed using Advanced Cell Diagnostics (ACD) RNAScope 2.5 LS Duplex Reagent Kit (Cat# 322440, ACD), RNAScope LS 2.5 Probe- Mm-Fos (Cat# 316928, ACD), and RNAScope 2.5 LS Probe- Mm-Crh-C2 (Cat# 316098-C2, ACD). Positive (RNAScope 2.5 LS Duplex Control Probes [PPIB-C1, Polr2A-C2]-Mm) and negative (RNAScope 2.5 LS Duplex Negative Control Probe [DapB-C1, DapB-C2]) controls were performed in parallel (ACD). Slides were processed as previously described (65).

### Drugs Studies.

For mouse study 5, cohorts of adult *Gdf15*^*−/−*^ mice and wild-type littermate control mice were injected (i.p.) with either LPS (Cat# L2880, Sigma) at 0.05 to 0.5 mg/kg or vehicle (phosphate-buffer saline [PBS]) and killed by cervical dislocation. For mouse study 6, cohorts of adult *Gdf15*^*−/−*^ and wild-type (C57BL/6J) control mice were infected with *E. coli* and samples were collected 4 h later. *E. coli* infections were performed as previously described (66). After 4 h, mice were killed by cervical dislocation. For mouse studies 7 and 8, cohorts of adult *Gdf15*^*−/−*^ mice and wild-type littermate control mice were used. A total of 10 mg/kg of cisplatin (Cat# NDC 0703-5747-11, Teva) or saline was administered via i.p. injection as a single dose at 9 AM. After 6 h of treatment, mice were killed by CO_2_ inhalation. For mouse studies 9 through 11, adult cohorts of *Gdf15*^*−/−*^ mice, as well as wild-type littermate control mice, were used. The animals were allocated to the experimental groups, matching body weight and age. Tunicamycin 0.1 mg/kg (Cat# T7765-5MG, Sigma) or vehicle (5% dimethyl sulfoxide [Sigma] in PBS) were injected i.p. at 8 to 9 AM. After 6 h of treatment, mice were killed. Blood was analyzed as described above.

### RNA Isolation/cDNA Synthesis for qPCR.

For mouse studies 7 through 10, total liver RNA (RNA) was extracted, purified, and analyzed as previously described (20).

### Quantification and Statistical Analysis.

For the mouse studies, results were statistically analyzed using Student’s *t* test or an analysis of variance (ANOVA) test using Prism 9 (GraphPad Software, Inc). For the rat study, differences were estimated from a repeated measurements model with baseline assessment as a covariate. For the human study, repeated measures with a post hoc Dunnett’s test were used to compare each time point with baseline.

## Supplementary Material

Supplementary File

## Data Availability

Further information and requests for resources and reagents should be directed to and will be fulfilled by the lead contact, Stephen O’Rahilly (so104@medschl.cam.ac.uk). For requests regarding the anti-GFRAL antibody used in this work please contact E-Chiang.Lee@kymab.com. This study did not generate/analyze new datasets/codes. All study data are included in the article and/or *SI Appendix*.
