# Supplementary material for: Activation of the hypothalamic–pituitary–adrenal axis by exogenous and endogenous GDF15
Source: Proc Natl Acad Sci U S A. 2021 Jun 29;118(27):e2106868118. doi: 10.1073/pnas.2106868118 (PMC8271778; doi:10.1073/pnas.2106868118)
Supplement: Supplementary File [file pnas.2106868118.sapp.pdf]

www.pnas.org

Supplementary Information for

Activation of the Hypothalamic-Pituitary-Adrenal axis by exogenous and endogenous GDF15

Irene Cimino[1, 15], Hanna Kim[2, 15], YC Loraine Tung[1], Kent Pedersen[3], Debra Rimmington[1], John A. Tadross[1,4], Sara N. Kohnke[1], Ana Neves-Costa[5], André Barros[5], Stephanie Joaquim[2], Don Bennett[6], Audrey Melvin[1], Samuel M. Lockhart[1], Anthony J. Rostron[7,8], Jonathan Scott[7], Hui Liu[9], Keith Burling[10], Peter Barker[10], Menna R. Clatworthy [11, 12, 13], E-Chiang Lee[9], A. John Simpson[7], Giles S.H. Yeo[1], Luís F. Moita[5, 14], Kendra K. Bence[2], Sebastian Beck Jørgensen[3,16], Anthony P. Coll[1, 16], Danna M. Breen[2, 16] and Stephen O'Rahilly[1,16, 17,]

[1]Metabolic Research Laboratories, Wellcome Trust-Medical Research Council Institute of Metabolic Science, University of Cambridge, Cambridge CB2 0QQ, UK;
[2]Internal Medicine Research Unit, Pfizer Inc, 1 Portland Avenue, Cambridge, MA, USA;
[3]Global Obesity and Liver Disease Research, Novo Nordisk A/S, Maaloev, Denmark
[4]Department of Pathology, University of Cambridge, Cambridge CB2 1QP, UK
[5]Innate Immunity and Inflammation Laboratory, Instituto Gulbenkian de Ciência, 2780-156 Oeiras, Portugal;
[6]Biostatistics, Early Clinical Development, Pfizer Inc, 1 Portland Street, Cambridge, MA, USA;
[7]Translational and Clinical Research Institute, Newcastle University, Newcastle upon Tyne, UK;
[8]Integrated Critical Care Unit, Sunderland Royal Hospital, South Tyneside and Sunderland NHS Foundation Trust
[9] The Bennet Building (B930), Babraham Research Campus, Kymab Ltd., Cambridge CB22 3AT UK
[10]Cambridge University Hospitals NHS Foundation Trust, Cambridge, UK;
[11]Molecular Immunity Unit, Department of Medicine, University of Cambridge, Cambridge, UK;
[12] Cambridge Institute of Therapeutic Immunology and Infectious Diseases, University of Cambridge, Cambridge, UK;
[13]Cellular Genetics, Wellcome Sanger Institute, Hinxton, UK.
[14] Instituto de Histologia e Biologia do Desenvolvimento, Faculdade de Medicina, Universidade de Lisboa, 1649-004 Lisboa, Portugal.

[15] These authors contributed equally
[16] Senior author
[17] Lead Contact
* correspondence: so104@medschl.cam.ac.uk (S.O.R.)

SI Methods:

Mice. Pfizer Inc. Mouse Studies 1-2*,* adult wild-type male mice (C57BL/6N; 14 - 16 weeks old) were obtained from Taconic Farms Inc. (Rensselaer, NY, USA). Mouse Studies 7, 8, 10, cohorts of *Gdf15*$^{-/-}$mice, and wild-type littermate control mice (22-26 weeks old), were obtained from a breeding colony at Jackson Laboratories (Farmington, CT). *Gdf15*$^{-/-}$mice were originally generated by the CRISPR/Cas9 system (C57BL/6J background). Mice were housed individually in Innovive cages (Innorack® IVC Mouse 3.5) under a standard 12 h light: dark cycle (06:00 h: 18:00 h) at standard housing temperatures (22 ± 1°C) or thermoneutral conditions (27 ± 1°C) with humidity-controlled environment. Mice were given *ad libitum* access to food and water. University of Cambridge. Mouse Studies 3, 4, 5, 12, 14 and 15*,* C57BL/6J male or female mice were purchased from Charles River (Charles River Ltd, Manston Rd, Margate, Kent, CT9 4LT) at 6-7 weeks of age. Mouse Studies 5 and 9 C57BL/6N-Gdf15tm1a(KOMP)Wtsi/H mice (herein referred to as "*Gdf15*$^{-/-}$ mice ") were bred in house from a line originally obtained from the MRC Harwell Institute. Mouse Study 11, cohorts of *Fgf21*$^{-/-}$, *Gdf15*$^{-/-:}$*Fgf21*$^{-/-}$ mice and wild-type littermates on a C57BL6/N background were obtained from het x het breeding pairs. *Fgf21*$^{-/-}$ mice were purchased from MMRRC. Mice were maintained in open vented cages in a 12 h light/12 h dark cycle (lights on 07:00–19:00), temperature-controlled (22°C) facility, with *ad libitum* access to food and water. This research was regulated under the Animals (Scientific Procedures) Act 1986 Amendment Regulations 2012 following ethical review by the University of Cambridge Animal Welfare and Ethical Review Body (AWERB). IGC. Mouse Study 6, *Gdf15*$^{-/-}$ mice were derived from the inbred C57BL/6 strain and were originally provided by S. Lee, Johns Hopkins University School of Medicine. *GDF15*$^{-/-}$and C57BL/6J control mice were bred and maintained under specific-pathogen free conditions at the Instituto Gulbenkian de Ciência on a 12 h light/12 h dark cycle, humidity 50 to 60%, ambient temperature 22 ± 2 °C, and with *ad libitum* access to food and water. All animal studies were performed in accordance with Portuguese regulations and approved by the Instituto Gulbenkian de Ciência Ethics Committee and Direção Geral de Alimentação e Veterinária (reference A002.2015).

Supplementary figure 1 (related to figure 1). Mouse corticosterone study

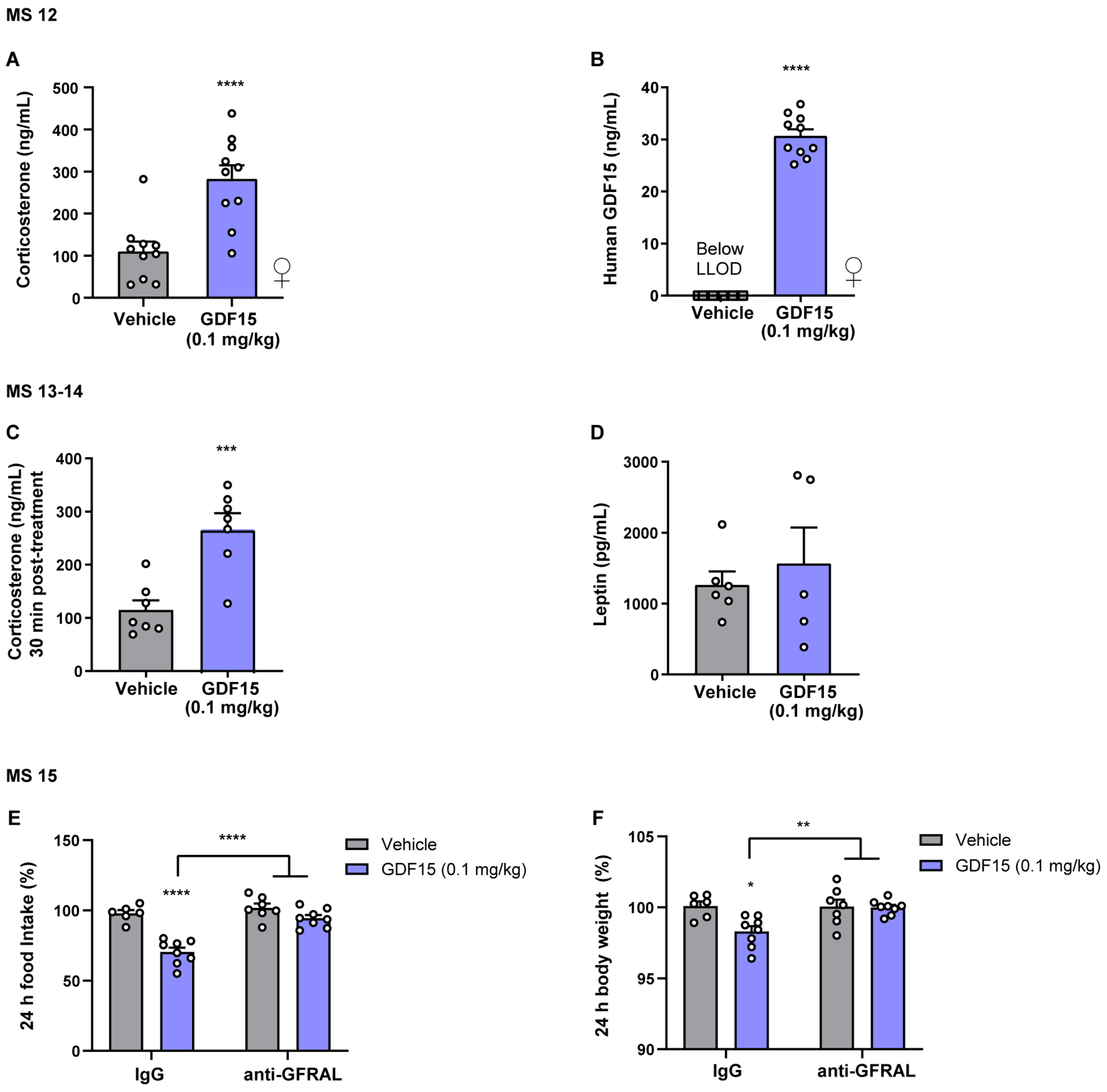

Fig. S1. GDF15 acute administration/leptin level/anti-GFRAL validation
(*A-B*) Mouse Study 12 (MS12): acute effect of human recombinant GDF15 administration on (*A*) endogenous corticosterone and human GDF15 (*B*) plasma concentration at 1 h.
(*C-D*) Mouse Study 13-14 (MS13-14): (*C*) Corticosterone serum level 30 min after human recombinant GDF15 injection at standard housing condition in mice. (*D*) Leptin serum concentration 1 h post human recombinant GDF15 injection in mice.
(*E-F*) Mouse Study 15 (MS15) Validation of GFRAL blocking antibody (anti-GFRAL) in mice. Percentage change in (*E*) food intake and (*F*) body weight in the 24 h following human recombinant GDF15 administration. Data are expressed as mean ± SEM, n = 6-8 per group. **p < 0.01, ***p < 0.001, ****p < 0.0001, for MS12-13-14 data were analysed by unpaired Student's t-test, for MS15 by ANOVA.

Supplementary figure 2 (related to figure 2). Rat corticosterone study

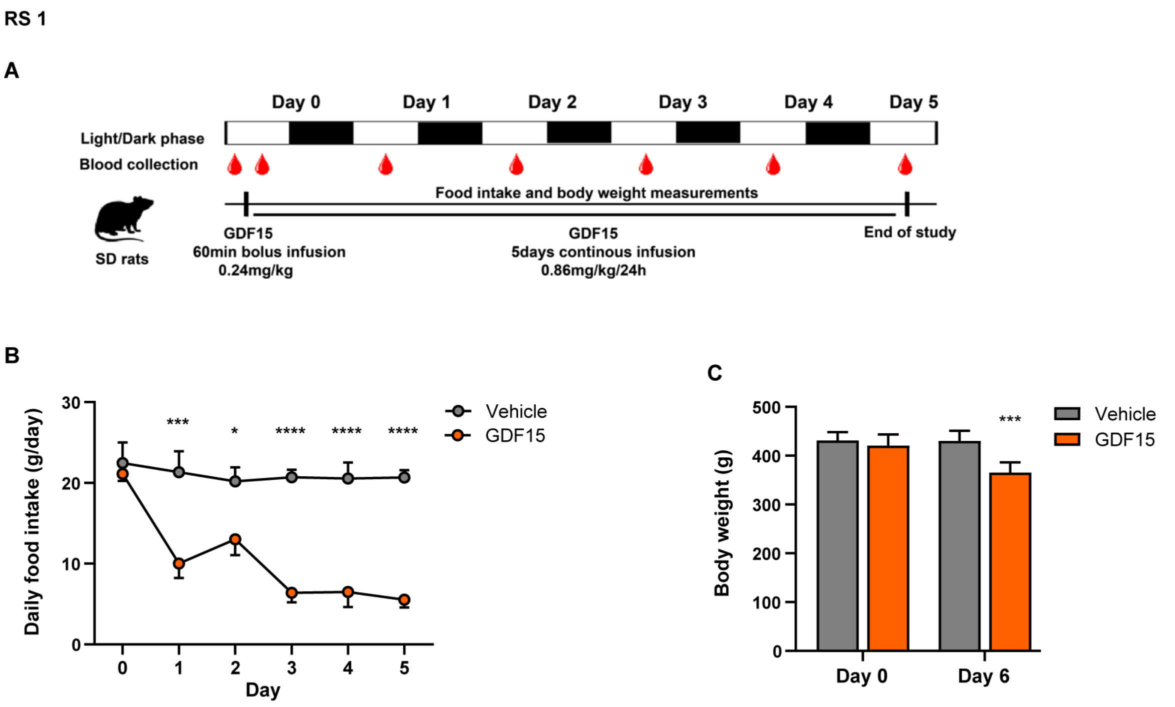

Fig. S2. GDF15 infusion validation Rat Study 1 (RS1): (*A*) Timeline of GDF15 administration and blood collection in the experiment (*B*) Daily food intake of rats continuously intravenous infused with vehicle or human GDF15. (*C*) Body weights prior to (day 0) and after continuous intravenous infusion (day 6) with vehicle buffer or human recombinant GDF15. Data are expressed as mean ± SEM, n = 6. *p < 0.05, ***p < 0.001, and ****p < 0.0001 by ANOVA.

Supplementary figure 3 (related to figure 3). Mouse corticosterone study

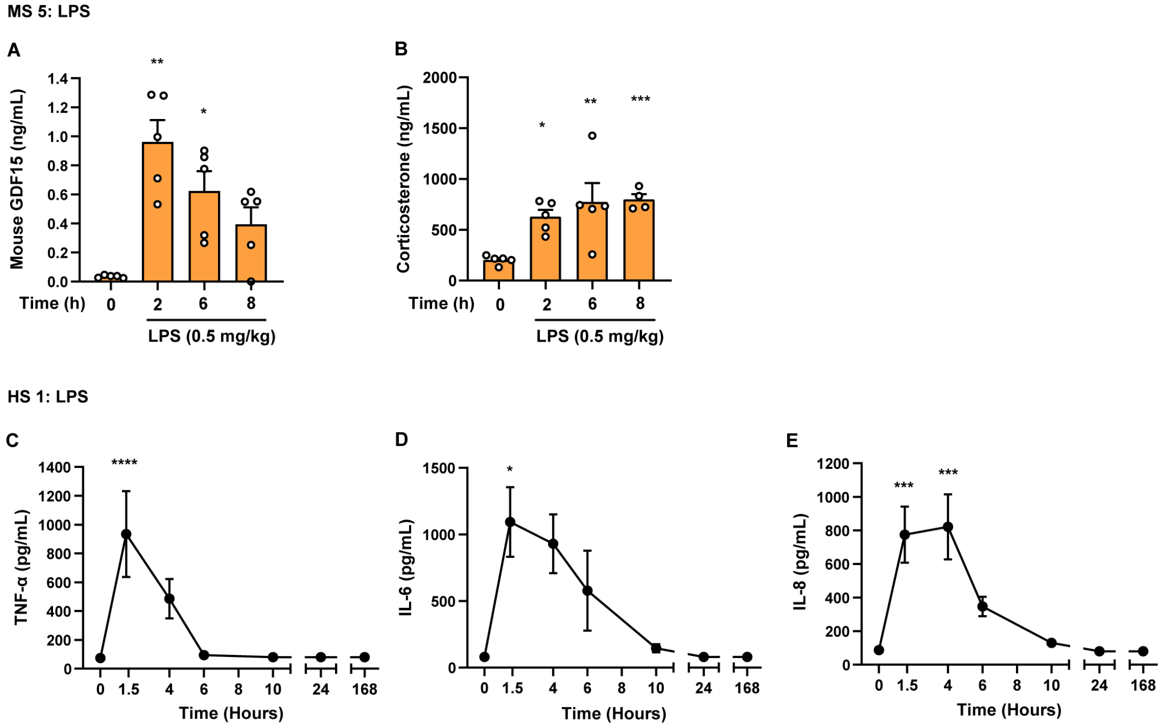

Fig. S3. Time course of GDF15 and corticosterone in the LPS model (A-B) Mouse Study 5 (MS5): Time course of (A) mouse GDF15 and (B) corticosterone serum concentrations at baseline (time=0) and at 2, 6 and 8 h after LPS (0.5 mg/kg) injection in mice. (C-E) Human Study 1 (HS1): Cytokine levels after LPS treatment in healthy human subjects. For MS5 data are expressed as mean ± SEM, n = 4-5 per group. $*p < 0.05$, $**p < 0.01$, $***p < 0.001$ by ANOVA. For HS1 data are expressed as mean ± SEM, n = 11. $*p < 0.05$, $***P<0.001$ by one-way repeated measures with post-hoc Dunnett's test to compare each timepoint with baseline.

Table S1. Cytokines – LPS mouse study

Cytokine (pg/mL)	0 h	2 h	6 h	8 h
**TNF-α**	16.8 ± 7.3	> 1258.0 [a]	494.6 ± 123.6 [b]	286.3 ± 46.5
**IFN-γ**	0.4 ± 0.2	4.4 ± 1.3	303.8 ± 101.5 [d]	296.4 ± 122.6
**KCGRO**	120.6 ± 32.6	> 3660.0 [a]	3099.0 ± 561.4 [a]	> 3660.0 [a]
**IL-1β**	1.6 ± 0.7	118.6 ±11.3 [a]	36.9 ± 7.92 [c]	37.2 ± 2.2 [d]
**IL-2**	< 1.2	8.9 ± 1.0 [a]	2.3 ± 0.8	2.9 ± 0.7
**IL-4**	< 0.3	< 0.3	< 0.3	< 0.3
**IL-5**	3.0 ± 0.9	21.3 ± 3.0	33.0 ± 1771 [c]	49.4 ± 12.21 [b]
**IL-6**	26.0 ± 11.6	> 9460 [b]	7689.0 ± 1771 [c]	8219.0 ± 1241.0 [c]
**IL-10**	13.0 ± 1.4	3474.0 ± 1812	479.8 ± 150.1	296.8 ± 23.6
**IL12p70**	< 40.0	476.2 ± 53.2 [a]	119.0 ± 36.8	136.3 ± 50.1

Data are expressed as mean ± SEM, a ****P<0.0001, b ***P<0.001, c **P<0.01, d *P<0.05

Table S1. Cytokines – LPS mouse study. Time course of mouse cytokine's concentrations at baseline (time=0) and at 2, 6 and 8 h after LPS (0.5 mg/kg) injection in mice.

Table S2. Cytokines – Tunicamycin mouse study at thermoneutrality

Cytokine (pg/mL)	Wild-type + veh	Wild-type + tun	*Gdf15* $^{-/-}$ + veh	*Gdf15* $^{-/-}$ + tun
**TNF-α**	6.9 ± 0.4	6.0 ± 0.7	6.8 ± 0.5	10.1 ± 0.9 [b]
**IFN-γ**	0.4 ± 0.0	0.3 ± 0.1	0.4 ± 0.0	0.5 ± 0.1
**KC/GRO**	40.9 ± 4.7	44.6 ± 6.7	45.2 ± 5.5	78.5 ± 13.5 [c]
**IL-1β**	< 0.3	< 0.3	< 0.3	< 0.3
**IL-2**	< 1.4	< 1.4	< 1.4	< 1.4
**IL-4**	< 0.1	< 0.1	< 0.1	< 0.1
**IL-5**	4.6 ± 0.4	3.2 ± 0.2	5.1 ± 0.8	4.1 ± 0.6
**IL-6**	< 1.4	2.5 ± 0.7	< 1.4	< 1.4
**IL-10**	183.0 ± 26.3	161.2 ± 15.09	200.1 ± 6.9	181.8 ± 6.0
**IL12p70**	131.2 ± 9.7	218.8 ± 22.1 [b]	133.3 ± 12.1	289.2 ± 24.2 [a]

Data are expressed as mean ± SEM, a ****P<0.0001, b **P<0.01, c *P<0.05

Table S2. Cytokines – Tunicamycin mouse study at thermoneutrality. Cytokines concentration after 6 h tunicamycin injection in wild-type and Gdf15-/- mice.
